# *Euclea divinorum* Hiern: Chemical Profiling of the Leaf Extract and Its Antioxidant Activity In Silico, In Vitro and in *Caenorhabditis elegans* Model

**DOI:** 10.3390/metabo12111031

**Published:** 2022-10-27

**Authors:** Hanin A. Bogari, Rasha M. H. Rashied, Mohamed A. O. Abdelfattah, Rania T. Malatani, Roaa M. Khinkar, Rawan H. Hareeri, Michael Wink, Mansour Sobeh

**Affiliations:** 1 Department of Pharmacy Practice, Faculty of Pharmacy, King Abdulaziz University, Jeddah 21589, Saudi Arabia; 2 School of Life and Medical Sciences, University of Hertfordshire Hosted by Global Academic Foundation, New Administrative Capital, Cairo 11835, Egypt; 3 College of Engineering and Technology, American University of the Middle East, Kuwait; 4 Department of Pharmacology and Toxicology, Faculty of Pharmacy, King Abdulaziz University, Jeddah 21589, Saudi Arabia; 5 Institute of Pharmacy and Molecular Biotechnology, Heidelberg University, Im Neuenheimer Feld 364, 69120 Heidelberg, Germany; 6 AgroBioSciences, Mohammed VI Polytechnic University, Ben-Guerir 43150, Morocco

**Keywords:** *Euclea divinorum* Hiern, antioxidant, *Caenorhabditis elegans*, molecular docking, drug discovery, industrial development

## Abstract

*Euclea divinorum* Hiern is a medicinal plant widely distributed in the northeast parts of South Africa. This plant has been used to treat miscarriage and to alleviate gastrointestinal problems. It can also be used externally for the treatment of ulcers and gonorrhea. In this study, we investigated the phytochemical composition of *E. divinorum* leaf extract using LC-MS and explored its antioxidant properties in vitro and in vivo. The total polyphenolic content of the extract was determined by the Folin–Ciocalteu method. DPPH and FRAP assays were employed to confirm the plant’s antioxidant potential in vitro. A survival assay in the *Caenorhabditis elegans* model was used to evaluate the extract’s ability to counteract juglone-induced oxidative stress. Moreover, a docking study was performed for the extract’s metabolites, in order to predict possible molecular targets that could explain the antioxidant effect of the plant on a molecular level. This in silico approach was accomplished on three different proteins; xanthine oxidase enzyme, heat shock protein 90 (Hsp90), and induced nitric oxide synthase (iNOS). Docking scores of the resulting poses and their interactions with binding sites’ residues were explored for each protein and were compared to those of simultaneously docked respective co-crystallized and reference substrates. The extract furnished promising antioxidant activities in vitro and in vivo in the *C. elegans* model that might be attributed to the presence of 46 compounds, which showed several interactions and low binding scores with the tested enzymes. In conclusion, *E. divinorum* is a promising, safe, and effective antioxidant candidate that could be used to ameliorate oxidative stress-related disorders.

## 1. Introduction

Reactive oxygen species (ROS), include mainly hydrogen peroxide (H_2_O_2_), singlet oxygen (^1^O_2_), superoxide (O_2_^•−^), and hydroxyl (^•^OH) radicals. Generally, ROS are side products of the different biological processes that take place in aerobic organisms, such as cellular respiration, protein phosphorylation, cellular differentiation, transcription factor activation, immune reactions, and programmed cell death [1,2]. Moreover, the generation of ROS is mediated by other endogenous and exogenous factors. In humans and superior mammals, mental stress, activation of immune cells, and aging are among the crucial endogenous factors that enhance ROS production in superior mammals. Exogenous factors include excessive exposure to heavy metals, environmental pollutants, radiation, smoking, alcohol intake, and some drugs such as gentamycin, cyclosporine, and tacrolimus [3,4].

Production of ROS mainly occurs in mitochondria, and enzymes associated with the respiratory chain, cytochrome P450 series, phagocytosis, and arachidonic acid metabolism are widely involved in the process [5]. The enzymes NADPH oxidase and xanthine oxidase play a key role in the production of the superoxide radical that in turn generates the hydrogen peroxide radicals via reactions mediated by some oxidase enzymes. The most reactive hydroxyl radical is produced when the superoxide radical reacts with hydrogen peroxide [6,7]. Generally, oxidation of the amino acid arginine by nitric oxide synthase results in the formation of the nitric oxide radical, which has an important role both physiologically and under pathological conditions [8]. On the other hand, production of ROS can take place via non-enzymatic reactions in which cellular organic compounds react with oxygen or become subjected to ionizing radiation [9].

Under normal conditions, our cells employ a powerful defense system to keep ROS levels under control. This system relies on several antioxidant enzymes that can scavenge different types of free radicals. These enzymes include glutathione peroxidase, catalase in peroxisomes, and superoxide dismutase in different compartments [10]. At low to moderate levels, ROS play a pivotal physiological role in living organisms. These include fighting pathogens, building some cellular structures, and regulating several intracellular signaling cascades. Particularly, nitric oxide is an important vasorelaxant that mediates proper blood flow within vessels. It is crucial for proper neural activity and is involved in thrombosis and non-specific host defense mechanisms [11,12].

However, excessive production of ROS causes a state of oxidative stress that is characterized by an imbalance between the ROS production and the cells’ ability to scavenge them. This condition wreaks havoc on many cellular structures such as membranes, nucleic acids, lipids, and proteins. Lipid peroxidation by ROS damages the cell membrane and lipoproteins, and results in the production of malondialdehyde and conjugated dienes that have been shown to be mutagenic and cytotoxic [13]. Upon the interaction between a protein and ROS, oxidation of specific amino acid residues occurs, followed by conformational changes in the protein structure that might lead to impairment or loss of protein activity [14]. Oxidative stress can also affect DNA as it mediates the oxidation of guanosine to 8-oxoguanosine, which can lead to point mutations and in addition to loss of epigenetic information [15]. Moreover, overexpression of heat shock proteins in response to oxidative stress leads to the activation of the NF-ĸB pathway and the subsequent production of the pro-inflammatory cytokines such as TNF, IL-1, and IL-6 due to interaction between these proteins and immune cells such as macrophages and dendritic cells [16]. Recent studies have pointed out that long-term interaction between heat shock proteins and immune cells may develop an immune response against the host’s antigens and promote a variety of autoimmune disorders [17]. Thus, oxidative stress can be responsible for the induction of several chronic diseases such as inflammation, cancer, Alzheimer’s, and diabetes, as well as speeding up cellular aging and acute pathologies [18]. Plant polyphenols have been proved to possess substantial antioxidant properties, which makes them promising safe and effective alternative drug candidates for treatment of several ailments [19].

The magic guarri or the diamond leaf, *Euclea divinorum* (Hiern), belongs to the family Ebenaceae that comprises four genera and about 855 species. The plant is a tropical evergreen shrub or small tree that grows up to around 18 m tall. It is widely distributed in southern and eastern Africa, especially in Ethiopia, Kenya, Mozambique, and S. Africa [20]. The leaves of *E. divinorum* are greyish green in color, with a leathery texture and wavy edges. The tree has small, cup-shaped creamy flowers and grey smooth bark that cracks upon aging. Traditionally, the fruits of the plant are used in the form of aqueous decoction as a mild laxative, while the dried roots are used as an infusion to treat jaundice, ulcers, miscarriage, arthritis, gastrointestinal disorders, headaches, and gonorrhea. The roots are also fermented and used in the beer industry. Other plant parts are used to treat anemia, diarrhea, and some other illnesses [20,21].

Phytochemical investigation of different *E. divinorum* extracts revealed several secondary metabolites that belong to various chemical classes, including polyphenols, tannins, alkaloids, saponins, sterols, terpenoids, and triterpenoids. Some individual compounds were also isolated from the plant extract. For instance, squalene, eicosane, and palmitic acid were isolated from the leaf extracts, 7-ethoxy coumarin, germanicol, and γ-sitosterol were isolated from the root bark extracts, while hexatriacontane and γ-tocopherol were isolated from the tender stem extracts. Secondary metabolites isolated from *E. divinorum* and its variable extracts have shown a wide array of pharmacological properties, including antiproliferative, antitumor, anti-inflammatory, analgesic, reno-protective, antimicrobial, antifungal, antiprotozoal, and insecticidal activities [20,21,22].

In this work, the phytochemical profile of the *E. divinorum* leaf methanol extract was identified using LC-MS/MS. The in silico antioxidant activity of some selected major metabolites identified in the extract was investigated via molecular docking on some molecular targets associated with oxidative stress, namely xanthine oxidase, inducible nitric oxide synthase (iNOS), and heat shock protein 90 (Hsp90). Moreover, the in vitro antioxidant potential of the extract was investigated by the Folin–Ciocalteu method for determination of the total phenolic content, DPPH, and FRAP assays, while its in vivo antioxidant activity was confirmed by survival assay in the *C. elegans* model. Finally, an extensive drug likeness analysis was performed on the major identified metabolites in the extract to explore some naturally occurring drug leads.

## 2. Materials and Methods

### 2.1. Plant Material and Extraction

*Euclea divinorum* Hiern was collected from Ethiopia in 2008. The identity of the plant was confirmed by DNA barcoding carried out in our laboratory using *rbcL* as a marker gene. The voucher specimen was deposited at the Department of Biology, Institute of Pharmacy and Molecular Biotechnology, Heidelberg University under the accession number P7393 at IPMB Heidelberg, Germany. The dried leaves (50 g) were ground and exhaustively extracted with 100% methanol at room temperature for an overall extraction period of 3 days. The combined extracts were filtered and reduced under vacuum at 40 °C till dry. The residue was frozen at −70 °C, and then lyophilized, yielding a fine dried powder (6.3 g).

### 2.2. LC-MS/MS

The LC system was ThermoFinnigan (Thermo Electron Corporation, Waltham, MA, USA) coupled with an LCQ-Duo ion trap mass spectrometer with an ESI source (ThermoQuest). The separation was achieved using a C18 reversed-phase column (Zorbax Eclipse XDB-C18, rapid resolution, 4.6 × 150 mm, 3.5 µm, Agilent, Santa Clara, CA, USA). A gradient of water and acetonitrile (ACN) (0.1% formic acid each) was applied from 5% to 30% ACN over 60 min with a flow rate of 1 mL/min with a 1:1 split before the ESI source. The samples were injected automatically using an autosampler surveyor ThermoQuest. The instrument was controlled by Xcalibur software (Xcalibur™ 2.0.7, Thermo Scientific, Waltham, MA, USA). The MS operated in the negative mode for better detection of the phenolic compounds with a capillary voltage of—10 V, a source temperature of 200 °C, and high-purity nitrogen as a sheath and auxiliary gas at a flow rate of 80 and 40 (arbitrary units), respectively. Collision energy of 35% was used in MS/MS fragmentation. The ions were detected in a full scan mode over a mass range of 50–2000 *m/z*.

### 2.3. In Silico Studies

Three proteins were downloaded from the protein databank (www.rcsb.org, accessed on 10 September 2022): xanthine oxidase (PDB ID:3NVY), heat shock protein (PDB ID: 5XRB), and induced nitric oxide synthase (PDB ID: 3NW2). Molecular Operating Environment software (MOE2020.0901) was used to perform the pre-docking preparation steps, the docking process, and to visualize potential poses and interactions. The proteins’ 3D structures were prepared using the structure preparation Quickprep panel in MOE. This allows protonation of structures, deletion of unbound water molecules, correction of structural errors as well as energy minimization. The compounds were downloaded from PubChem as individual molecules (.sdf files), then compiled into one database. The respective co-crystallized and reference ligands for each protein were inserted using the MOE builder tool and added to the database for the purpose of docking protocol validation. All ligands were prepared by database wash to adjust formal charges for strong acids and bases and to adjust bond lengths’ scales. Ligands were also checked for any structural bonding errors and were then adjusted to the most abundant tautomeric state using the Protomers panel. Atomic partial charges were set, and energies were minimized to a gradient of 0.0001 to ensure accurate subsequent calculations. The docking protocol was implemented using triangle matcher as a placement method, refinement was carried out using induced fit. Scoring of the potential docking solutions was carried out using the default London dG scoring function and the refined poses were scored using the default GBVI/WSA dG scoring function. The output files were explored, and the resulting poses were filtered according to their scores in addition to the interactions they exhibited with the amino acids of the binding site.

Molecular Operating Environment software is a product of the Chemical Computing Group. It is an integrated computer-aided molecular design software, which allows molecular simulations. In this research, the software was used for a structure-based design. MOE2020.0901 is fully functional on a research license at the University of Hertfordshire in Egypt.

As for the docking scores, the generated poses were first scored using London dG scoring function, refinement of the poses was implemented by induced fit to allow for ligand flexibility, the final energy was evaluated using the generalized Born solvation model (GB/VI) which is a forcefield-based scoring function that estimates the free energy of binding of the ligand. The scoring function consists of terms that represent average gain/loss of rotational and translational energy, coulombic electrostatic term, solvation electrostatic term, surface area weighted by exposure. The scoring function also accounts for van der Waals contribution to the free energy of binding.

### 2.4. Total Phenolic Content

The Folin–Ciocalteu method using a 96-well microplate was employed to determine the total polyphenolic content of the extracts. Firstly, the 96-well microplate was filled with 20 μL of 1 mg/mL of each extract, afterwards Folin–Ciocalteu reagent was added in the concentration of 10% (*v/v*, 100 μL), and the microplate was incubated at room temperature in the dark for 5 min. Then, 80 μL Na_2_CO_3_ (7.5% *w*/*v*) was added to the microplate with good mixing, followed by incubation for another 30 min at room temperature in the dark. A UV–Vis spectrophotometer at 765 nm (Jenway, Model 6305) was used to measure the absorbance. Gallic acid was used as a standard to prepare a calibration curve and the TPC was presented as mg of gallic acid equivalents per mg of extract (mg GAE/g). The experiment was carried out in triplicate.

### 2.5. Antioxidant Activity In Vitro

#### 2.5.1. DPPH Radical Assay

The radical scavenging activity was assessed using the stable free radical 2,2-diphenyl-1-picrylhydrazyl (DPPH•). The assay was performed according to the standard technique described by Blois (1958) with some modifications to a 96-well microplate. In brief, 100 µL of DPPH solution (200 µM) was added to 100 µL of the extract with concentrations ranging from 50–1.25 µg/mL. In the dark at room temperature, the samples were incubated for 30 min. The absorbance was measured at 517 nm. The ability of the samples to scavenge the DPPH radicals was calculated according to the following equation: DPPH scavenging effect (%) = [(A0 − A1)/A0] × 100 where A0 represents the control absorbance, and A1 represents the absorbance of the extract. All measurements were performed in triplicate. The IC_50_ value (µg extract/mL) was estimated using GraphPad software. Quercetin was used as a positive control.

#### 2.5.2. Ferric Reducing Antioxidant Power Assay

The ferric reducing antioxidant power (FRAP) assay was carried out according to the previously reported procedure with minor modifications. Each sample was dissolved in methanol to prepare the stock solution (1 mg/mL) [23]. Briefly, the working FRAP reagent was prepared freshly by mixing 300 mM acetate buffer (pH 3.6), a solution of 10 mM 2,4,6-tripyridyl-s-triazine (TPTZ) in 40 mM hydrochloric acid, and 20 mM ferric chloride at 10:1:1 (*v*/*v*/*v*). Then, 20 µL of each extract was mixed with 180 µL FRAP reagent in wells of 96-well plates. The mixture was then incubated for 6 min at 37 °C, and the absorbance was measured at 595 nm in a microplate reader (Biochrom Asys UVM 340). Appropriate blanks of plant extract and of FRAP reagent lacking TPTZ (to correct the colors of the extracts) were run, together with quercetin (in methanol), and ferrous sulfate heptahydrate (FeSO_4_.7H_2_O) was used as a standard. FRAP activity was calculated as ferrous equivalents (FE), the concentration of extract/quercetin which produced an absorbance value equal to that of 1 mM FeSO_4_. Quercetin was used as a positive control.

### 2.6. Antioxidant Activity In Vivo

#### 2.6.1. *Caenorhabditis elegans* Strains and Maintenance

Nematodes were maintained under standard conditions (20 °C, on nematode growth medium (NGM), fed with living *E. coli* OP50). Age-synchronized cultures were obtained by sodium hypochlorite treatment of gravid adults; the eggs were kept in M9 buffer for hatching and larvae obtained were subsequently transferred to S-media seeded with living *E. coli* OP50 (D.O_600_ = 1.0). The wild type (N2) was obtained from the *Caenorhabditis* Genetic Center (CGC), University of Minnesota, USA.

#### 2.6.2. Survival Assay under Juglone-Induced Oxidative Stress

Early larval stage (L1) wild type worms were treated with different concentrations of the extract (100 and 200 µg extract/mL), except the control group, and maintained at 20 °C for 48 h in S-medium; then, a single dose with a final concentration of 80 µM juglone was added to the media. The survivors were counted after 24 h after the addition of the pro-oxidant naphthoquinone juglone. The results are presented as a percentage of live worms.

### 2.7. Statistical Analysis

All data are expressed as mean ± SEM. Statistical analysis was performed by ANOVA followed by Tukey post hoc test with GraphPad Prism 6 (GraphPad, CA, USA). A *p*-value < 0.05 was considered statistically significant.

## 3. Results and Discussion

### 3.1. Chemical Composition

**Figure 1:**
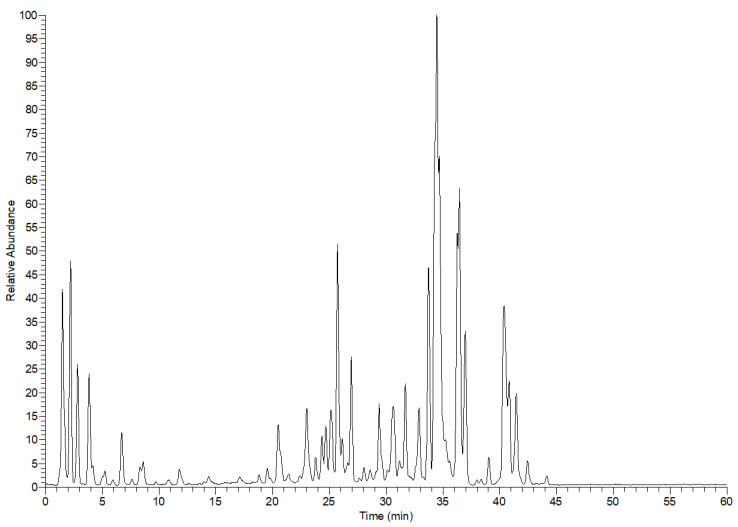
*E. divinorum* leaf extract profile in the negative ion mode by LC-MS.

Altogether, 46 compounds were tentatively characterized from the leaf extract of *E. divinorum.* These compounds belong to organic acids, simple phenolic acids and their glycosides and sulfate derivatives, flavonoids, and mono-, dimer-, and trimer- proanthocyanidins, Table 1 and Figure 1. The prefix (*epi*) indicates that the stereochemistry of these compounds is not resolved.

### 3.2. In Silico Results

In the current work, we investigated, using in silico molecular docking, the potential of the major extract’s components to interfere with three target proteins with known roles in mediating oxidative stress and the consequent inflammatory response, namely xanthine oxidase, heat shock protein 90, and inducible nitric oxide synthase.

The molecular docking served as a tool to explore which constituents of the extract are expected to demonstrate good fitting, and thus good binding affinity, towards the binding sites of the molecular targets. The binding affinity of the docked compound was estimated by the docking score parameter, which quantifies the stability of the binding between the docked pose and the amino acids lining the binding site. The top poses are further explored to confirm their interaction with crucial reported amino acids. For a top scoring compound, the mere presence of interactions with the crucial amino acids in the binding site does not suffice; rather, a top hit candidate must show minimum docking score relative to the reference drugs and the other compounds in the extract. Scoring stems from enthalpy and entropy changes, and it depends on good electronic complementarity as well as good steric complementarity and the 3D orientation of interacting partners plays a role in mediating interactions.

Xanthine oxidase is an enzyme that mediates the generation of uric acid via the oxidation of hypoxanthine to xanthine that is further oxidized by the enzyme to uric acid. The enzyme is classified among the oxidoreductases owing to its ability to produce some reactive oxygen species, such as superoxide and hydrogen peroxide. The regulation of the enzyme’s activity via xanthine oxidase inhibitors is expected to treat hyperuricemia, decrease oxidative stress, and attenuate the consequent pathophysiological inflammatory responses. The crystal structure of bovine xanthine oxidase complexed with the natural flavonoid inhibitor quercetin was explored (PDB ID: 3NVY). It is worth mentioning that the bovine form of the enzyme shares 90% sequence similarity to the human xanthine oxidase and the amino acids involved in the binding are strictly conserved [24]. Polar groups of quercetin form hydrogen bonds to amino acids Glu802, Arg880, and Thr1010. The two fused rings of quercetin are sandwiched between the phenylalanine residues Phe914 and Phe1009. This resembles the binding modes of most of the substrates of xanthine oxidase. Additional van der Waals interactions are reported with the hydrophobic residues Leu873, Leu1014, Leu648, Val1011, Phe1013 [24].

The docked compounds revealed docking scores on xanthine oxidase that spanned a range of −4.18 to −8.30 kcal/mol (Table 2). Among these compounds, three docked candidates exhibited better binding scores than the docked co-crystallized ligand quercetin and the reference inhibitor febuxostat. These three compounds are 23, 31, and 33, which displayed docking scores of −8.30, −7.70, and −7.61 kcal/mol, respectively, in comparison to −5.81 and −7.55 kcal/mol for quercetin and febuxostat. Examining the docked poses, quercetin was found to preserve hydrogen bonding to the residue Glu802 as well as the hydrophobic interaction with Val1011. Docked febuxostat displayed polar interactions of the carboxylate moiety with Arg880 and Thr1010. The thiazole ring of febuxostat interacts via hydrogen bonding to Glu802 and Thr1010 and also via pi–pi stacking to the residue Phe914.

Heat shock proteins are closely related to oxidative stress and their overexpression during this state mediates inflammatory and immunomodulatory responses.

Heat shock protein 90 (Hsp90) is a homodimer of which each polypeptide chain consists of three regions: N-terminal ATP-binding domain, a middle domain, and a C-terminal domain. It is considered an attractive target due to its control of the cellular activity of several regulatory and signal transduction proteins [25]. The crystal structure of the N-domain of Hsp90 was determined by X-ray crystallography complexed to the inhibitor FJ5 and deposited in the protein databank (ID: 5XRB). The inhibitor FJ5 and well-known natural product inhibitor radicicol were both docked simultaneously with the test compounds, serving as co-crystallized ligand and reference ligand, respectively. Reported interactions of several inhibitors from different classes to the ATP binding site of this protein include interactions with the highly conserved residue Asp93, as well as interactions with Leu48, Asn51, Asp93, Gly97, and Asp102 [26,27].

The docked compounds displayed binding modes within the ATP domain that reproduced the reported interactions, their docking scores range between −4.05 and −9.81 kcal/mol. Meanwhile, docking the inhibitors FJ5 and radicicol into the protein’s binding site affords docking scores of −8.27 and −6.77 kcal/mol, respectively. The co-crystallized ligand FJ5 validated the docking protocol since it exhibited a similar binding mode to the co-crystallized mode preserving the interactions with Ser52, Asp93, and Thr184. The reference inhibitor radicicol also displayed a hydrogen bond interaction with the amino acid Asp93. Seven out of the thirty-six docked test compounds (7, 15, 22, 23, 32, 38, and 39) exhibited better docking scores than both the co-crystallized and reference ligands. Docking results on Hsp90 are summarized in Table 3.

A high level of ROS during oxidative stress induces the enzyme inducible nitric oxide synthase (iNOS) and thus increases the production of NO, which in conjunction with other ROS contributes greatly to oxidative stress. The enzyme is a NOS isoform expressed due to pro-inflammatory factors. Overproduction of iNOS leads to high NO levels, and is correlated with various diseases, such as sepsis and altered immune response. Consequently, the development of iNOS inhibitors is desirable since they are associated with the alleviation of various pathologies, such as diabetes and neurologic and respiratory diseases [28]. Murine iNOS was reported to show 89% sequence similarity to human iNOS and amino acids involved in the binding are highly conserved [29]. The enzyme was deposited in the protein databank complexed with an imidazo[4,5-b]pyridine derivative (MPW), PDB ID: 3NW2. The ligand accommodates a hydrophobic pocket formed of Val346 and Phe363 and displays a salt bridge with the polar residue Glu371. Another hydrogen bond is displayed with the residue Arg382. Moreover, hydrophobic contacts with Trp84, Met114, Ser256, Arg260 have been implicated by other iNOS inhibitors [29].

In this study, MPW was re-docked into the iNOS active site. Additionally, the reported selective inhibitor *N*-(3-(aminomethyl)benzyl)acetamidine (1400 W) was chosen to be docked simultaneously with our test compounds [30]. The docked MPW adopts a conformation in the active site in which its aromatic rings display hydrophobic interactions with Ile195 and Trp366. Meanwhile, the refence inhibitor 1400 W shows hydrogen bonding to amino acids Asn364 and Glu371. The docked natural compounds demonstrated good affinities to the iNOS enzyme which was revealed by the docking scores ranging between −3.83 and −11.05 kcal/mol, of which 14 compounds displayed better scores than both co-crystallized and reference ligands. The docking results on iNOS are summarized in Table 4.

In an attempt to further validate our docking procedure, all compounds achieving better scores than both the co-crystallized and reference inhibitors on each of the three proteins were further re-docked twice and average scores of the three docking trials were calculated. Simultaneously, the co-crystallized and reference substrates for each protein were re-docked. The results of the docking trials are found in Table 5.

**Figure 2:**
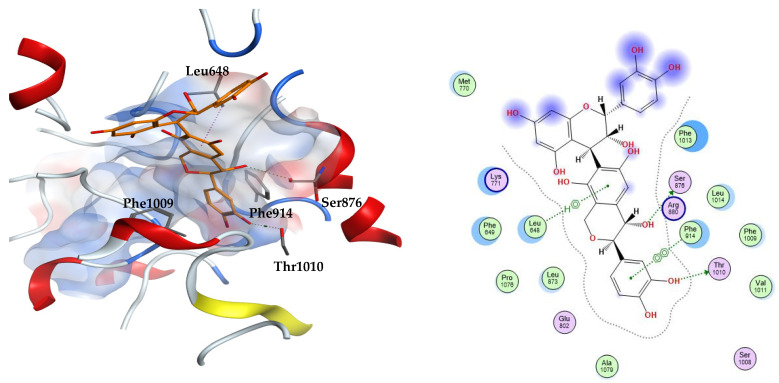
The 3D (**left**) and 2D (**right**) interactions of compound 23 (epicatechin(4b->6)catechin) with xanthine oxidase binding cavity.

From the three docking trials, the best two ligands for each protein were explored. Compounds **23, 31** were the top scorers on xanthine oxidase with average docking scores of −8.30 and −7.65 kcal/mol, respectively. The average scores of quercetin and febuxostat were −5.82 and −7.54 kcal/mol, respectively. The interactions afforded by the docked pose of the highest scoring candidate **23** were investigated. The compound established hydrogen bonds to the residues Ser876 and Thr1010, arene–hydrogen interaction with Leu648, and pi–pi stacking with Phe914. Compound **23** appears in an orientation sandwiched between the two aromatic residues Phe914 and Phe1009 at the binding site of xanthine oxidase, similar to the binding mode of the co-crystallized substrate, quercetin. This demonstrates the stability of this ligand–enzyme complex (Figure 2).

**Figure 3:**
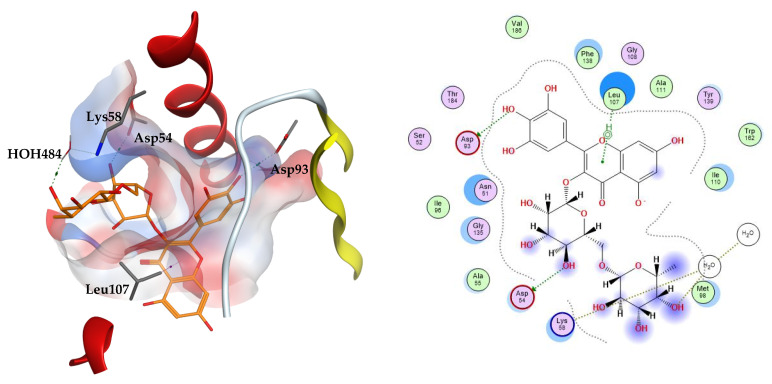
The 3D (**left**) and 2D (**right**) interactions of myricetin rutinoside with Hsp90 binding cavity.

The two top scoring ligands on Hsp90 were compounds **32** and **39** with average docking scores of −9.92 and −9.64 kcal/mol, respectively. The average scores of docked FJ5 and radicicol were −8.47 and −6.16 kcal/mol, respectively. The best scoring candidate, compound **32**, displayed arene–hydrogen interaction with Leu107, direct hydrogen bonding to Asp54 and Asp93, in addition to indirect hydrogen bonding to the amino acid Lys58 through a structural water molecule, Figure 3.

**Figure 4:**
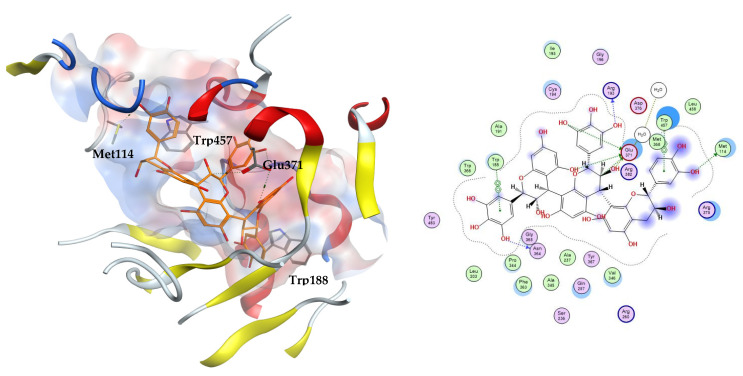
The 3D (**left**) and 2D (**right**) interactions of prodelphinidin C2 with iNOS binding cavity.

The three docking trials carried out on iNOS enzyme revealed that the top two scoring compounds were compounds **22** and **23** with average docking scores of −11.49 and −9.90 kcal/mol, respectively. These docking scores showed the stability of the complexes between our docked ligands and the induced nitric oxide synthase enzyme compared to MPW and 1400 W with average docking scores of −6.65 and −5.68 kcal/mol, respectively. The highest scoring compound **22** is able to fit inside the iNOS binding cavity and perform several hydrogen bonds to the key residue Glu371. Additionally, the compound interacts with the hydrophobic amino acids Met114, Trp188, and Trp457, Figure 4.

### 3.3. Drug Likeness Analysis

In order to investigate the potential of the compounds identified in *E. divinorum* extract to serve as drug leads, we ran an extensive physicochemical property and drug likeness analysis based on the most used measures, namely Lipinski, Veber, Egan, Ghose, and Muegge.

Lipinski set five criteria for a compound to act as a drug lead: a molecular weight of no more than 500, no more than 5 H-bond donors, no more than 10 H-bond acceptors, and log-P of no more than 5. Compounds showing not more than one violation of these criteria can be promising drug candidates. Veber considered two other criteria for a compound to show good oral absorption: topological polar surface area (TPSA) and the number of rotatable bonds. TPSA is the sum of the surfaces of all the polar atoms in a given compound. According to Veber, a compound with TPSA ≤ 140 A^2^ and less than 10 rotatable bonds can be absorbed well after oral administration and able to reach its molecular target. Egan stated that compounds with TPSA less than 132 A^2^ and log-P value between −1 and 6 can show good orally bioavailability and serve as drug leads. The bioavailability score and sp^3^ carbon fraction are two other measures of oral bioavailability. The former indicates the ability of a compound to be more than 10% bioavailable in absorption assays, while the latter is an indication of the compound’s flexibility degree. Highly flexible compounds do not usually have good oral bioavailability as they are less planar and have a complex 3D shape. Generally, compounds complying with the Lipinski rule with a bioavailability score of 0.55–0.85 and Csp^3^ score between 0.25 and 1 are considered orally bioavailable [31].

Muegge utilized simple structural rules to set a pharmacophore point filter to distinguish drug-like from non-drug-like compounds. According to this filter, a possible drug-like candidate should have log-P value between −2 and 5, a maximum TPSA of 150 A^2^, less than 5 H-bond donors, less than 10 H-bond acceptors, less than 15 rotatable bonds, less than 7 rings, at least 4 carbon atoms and 1 heteroatom, and a molecular weight ranging from 200 to 400. Finally, Ghose based his drug likeness criteria on four parameters: a molecular weight between 160 and 480, a log-P value between −0.4 and 5.6, a molar refractivity between 40 and 13o, and a total atom number between 20 and 70 [31].

Table 6 shows that among the investigated compounds, 10 showed only one or no violation of all the drug likeness criteria utilized in the physicochemical properties’ analysis. These compounds are 14, 17, 21, 24, 27, 29, 30, 35. All of the eight compounds were shown to be of high GI absorption (except 14, 17, and 30), good synthetic accessibility, and not able to inhibit the different cytochrome enzyme systems; thus, there was a low chance for possible drug–drug interactions, and they were not able to penetrate the blood–brain barrier; thus a low possibility for central adverse reactions, Table 7. In view of this thorough analysis, these compounds can be presented as good drug leads of natural origin.

Moreover, drug likeness analysis showed that six compounds only are expected to inhibit Cyp3A4 only among the different cytochromes investigated. The rest of the compounds did not show potential to interfere with any of the cytochrome enzymes. This indicates that the extract’s components might show minimal potential drug–drug and drug–food interactions.

### 3.4. In Vitro Results

The in vitro antioxidant activity of *Euclea divinorum* extract was initially investigated by DPPH and FRAP assays. The extract showed substantial antioxidant potential in both assays. The extract’s antioxidant activities in these assays were comparable to the quercetin reference inhibitor as shown in Table 8. The powerful antioxidant properties of the extract could be due to the high phenolic content that was found to be 132 mg gallic acid equivalents (GAE)/g extract, Table 8.

### 3.5. In Vivo Results

**Figure 5:**
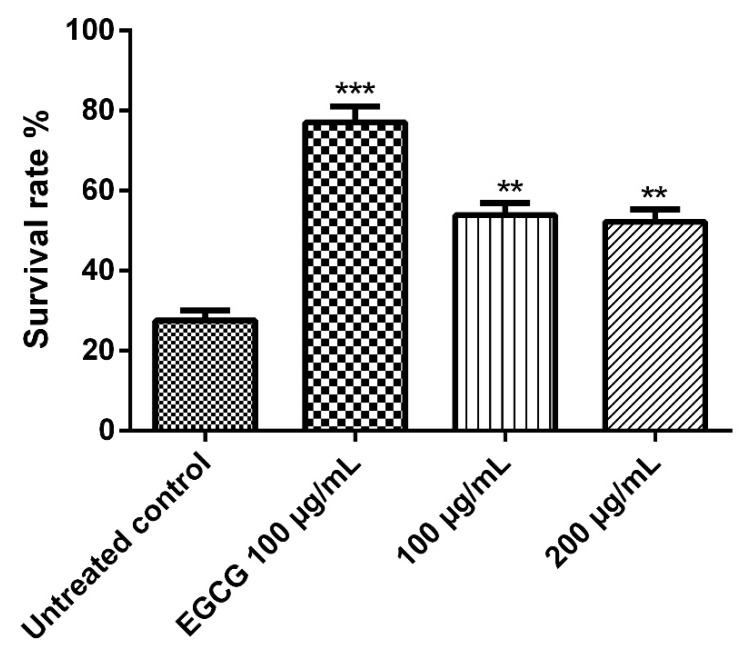
Survival rate of the worms under juglone (80 μM) treatment. Treatment of the worms with 100 and 200 μg/mL of *E. divinorum* extract on N2 nematodes. *E. divinorum* extract improved their resistance to oxidative stress and increased the survival rate to 56.29% and 50.00%, respectively, the positive control, EGCG (100 μg/mL), increased the survival rate to 76.00%. The survival rate of the juglone treatment alone was 26.32%. Significant differences relate to the control group at ** *p* < 0.01 and *** *p* < 0.001 by one-way ANOVA and Tukey’s post hoc test.

The nematode *C. elegans* model is a good model for studying some biological effects such as the antioxidant properties, where it shares lots of similarities, at the molecular level, with human systems despite being significantly simpler anatomically than humans, thus we employed it in the current work. Juglone is a powerful pro-oxidant that can be isolated from *Juglans regia* and an exposure to a dose of 80 µM of juglone induces substantial oxidative stress and some other cytotoxic effects. As shown in Figure 5, juglone treatment reduced the survival rate of the nematodes to 26.3%, however, *E. divinorum* extract counteracted the juglone-induced oxidative stress and increased the survival rate of the worms in a dose-dependent manner compared to the reference EGCG. It is worth mentioning that an aqueous bark extract from *Warburgia salutaris* with similar phytochemical composition to that of *E. divinorum* was able to increase the survival rate of the nematodes by virtue of counteracting the oxidative stress through decreasing the expression of HSP16 and enhancing the nuclear delocalization of DAF16, which is a forkhead transcription factor involved in cell cycle arrest, apoptosis, stress resistance, age modulation, and longevity [26].

## 4. Conclusions

Here, *E. divinorum* leaf extract furnished 46 compounds using LC-MS/MS. The major metabolites identified in the extract showed promising antioxidant activities in silico on three molecular targets associated with oxidative stress, namely xanthine oxidase, inducible nitric oxide synthase (iNOS), and heat shock protein 90 (Hsp90). The docking study suggested the formation of stable complexes between the extracts’ metabolites and the amino acids lining the binding sites of the said targets. The top scoring compounds were chosen based on average docking scores obtained in different trials, and they were found to retain the reported interactions with crucial amino acids. The compounds’ activities were validated in vitro via DPPH and FRAP assays, and in vivo by the survival assay in the *Caenorhabditis elegans* model. A drug-likeness analysis was performed for the extract’s metabolites to assess the oral bioavailability of the compounds and to exclude drug–drug interactions or CNS side effects. The in silico analysis along with the observed biological activities show the possibility of the top scoring compounds under study to serve as natural lead compounds for the development of other potent inhibitors of the three proteins and serve as antioxidants.

## Figures and Tables

**Table 1:** *E. divinorum* leaf extract composition by LC-MS.

No.	Rt	M-H	MS/MS	Proposed Names
**1**	1.58	191	127	Quinic acid
**2**	1.68	133	115	Malic acid
**3**	1.77	173	111	Shikimic acid
**4**	2.20	191	111	Citric acid
**5**	3.77	169	126	Gallic acid
**6**	4.07	331	169	Galloyl glucose
**7**	4.17	609	305	(epi)Gallocatechin-(epi)gallocatechin
**8**	5.90	609	305	(epi)Gallocatechin-(epi)gallocatechin
**9**	6.23	153	109	Dihydroxybenzoic acid
**10**	6.66	411	169, 331	Galloyl glucose sulfate
**11**	7.61	153	109	Dihydroxybenzoic acid
**12**	8.24	593	289	(epi)Gallocatechin-(epi)catechin
**13**	8.60	305	179	(epi)Gallocatechin
**14**	9.72	325	169	Gallic acid shikimate
**15**	10.73	593	289	(epi)Gallocatechin-(epi)catechin
**16**	11.71	365	153, 285	Dihydroxybenzoic acid pentosyl sulfate
**17**	11.91	285	153	Dihydroxybenzoic acid pentoside
**18**	14.02	295	163	Coumaric acid pentoside
**19**	14.70	137	93	*p*-Hydroxybenzoic acid
**20**	16.31	761	305, 609	(epi)Gallocatechin-(epi)gallocatechin gallate
**21**	17.06	183	169	Methyl gallate
**22**	18.89	897	305	Prodelphinidin C2
**23**	19.48	577	289	(epi)Catechin-catechin
**24**	20.41	289	179	(epi)Catechin
**25**	20.60	761	305, 609	(epi)Gallocatechin-(epi)gallocatechin gallate
**26**	20.76	353	191	Chlorogenic acid
**27**	22.35	333	289	(epi)Catechin carboxylic acid
**28**	24.43	319	193	Trihydroxy benzoyl ferulic acid
**29**	24.82	305	225	(epi)Gallocatechin
**30**	25.65	385	223	Sinapic acid glucoside
**31**	26.53	457	305	Epigallocatechin gallate
**32**	29.13	625	317	Myricetin rutinoside
**33**	29.44	441	289	(epi)Catechin gallate
**34**	30.63	479	317	Myricetin glucoside
**35**	30.78	279	163	Protocatechuic acid malate
**36**	31.95	449	317	Myricetin pentoside
**37**	33.71	449	317	Myricetin pentoside
**38**	34.48	463	317	Myricetin rhamnoside
**39**	35.16	609	301	Rutin
**40**	36.22	419	287	Eriodictyol pentoside
**41**	36.74	493	331	Methylmyricetin glucoside
**42**	38.33	433	301	Quercetin pentoside
**43**	40.39	447	301	Quercetin rhamnoside
**44**	40.77	287	259	Eriodictyol
**45**	41.48	419	287	Caffeoyl eriodictyol
**46**	42.46	317	179	Myricetin

**Table 2:** Docking scoring functions (kcal/mol) of *E. divinorum* metabolites with xanthine oxidase.

No. *	PubChem CID	Score	Interactions
**1**	6508	−4.18	Glu802, Thr1010, Ala1079
**2**	525	−4.92	Arg880, Thr1010, Val1011
**3**	8742	−5.38	Glu802, Arg880
**4**	311	−4.37	Glu802, Ser876, Arg880, Thr1010, Ala1078
**5**	370	−5.23	Glu802, Phe914, Thr1010
**6**	124021	−6.69	Ser876, Arg880, Phe914, Thr1010
**7**	13831068	−7.14	Leu648, Lys771, Arg880, Phe914, Phe1009, Thr1010
**9**	3469	−5.45	Glu802, Phe914, Phe1009, Thr1010
**10**		−7.22	Leu648, Lys771, Glu802, Arg880, Thr1010
**11**	19	−5.70	Glu802, Phe914, Thr1010, Ala1079
**14**		−6.94	Asn768, Lys771, Glu802, Phe914, Thr1010
**15**	11527214	−5.92	Leu648, Lys771, Ser876
**15′**	72193638	−7.05	Leu648, Phe649, Lys771, Phe914
**16**		−5.55	Arg880, Thr1010, Val1011
**17**		−6.88	Glu802, Arg880, Phe914, Ala1079, Glu1261
**19**	135	−5.18	Glu802, Phe914, Phe1009, Thr1010
**21**	7428	−5.51	Arg880, Phe914, Thr1010
**22**	71623698	−7.16	Ser876, Leu1014
**23**	131752345	−8.30	Leu648, Ser876, Phe914, Thr1010
**24**	72276	−6.40	Leu648, Phe914, Thr1010
**26**	1794427	−7.15	Lys771, Phe914, Thr1010
**27**		−6.51	Leu648, Glu802
**29**	72277	−6.81	Phe914, Thr1010
**30**	5280550	−7.06	Leu648, Lys771, Glu802, Val1011
**31**	65064	−7.70	Leu648, Glu802, Thr1010, Leu1014
**32**	44259428	−6.65	Lys771, Arg880, Thr1010
**33**	107905	−7.61	Leu648, Met770, Glu802, Thr1010
**34**	5318606	−6.79	Leu648, Lys771, Leu1014
**35**		−6.42	Lys771, Phe914, Phe1009
**37**	21477996	−6.86	Leu648, Lys771, Ser876
**37**	73208527	−5.92	Phe649, Lys771, Arg880, Thr1010
**38**	5352000	−6.54	Lys771, Glu802, Phe914, Phe1009, Thr1010, Pro1076
**39**	5280805	−6.62	Leu648, Lys771
**42**	5878729	−6.15	Phe914, Thr1010
**44**	440735	−6.80	Leu648, Glu802, Phe914, Phe1009, Thr1010
**46**	5281672	−6.49	Arg880, Phe914, Thr1010, Val1011
**Quercetin (control)**		−5.81	Glu802, Val1011
**Febuxostat (control)**		−7.55	Glu802, Arg880, Phe914, Thr1010

* Compound numbers from Table 1.

**Table 3:** Docking score (kcal/mol) and interactions of *E. divinorum* metabolites in the active site of heat shock protein 90 (Hsp90).

No. *	Score	Interactions
**1**	−5.16	Asn51, Ser52, Asp93, Gly97, Thr184
**2**	−4.05	Asn51, Asp93, Gly97, Thr184
**3**	−4.93	Asn51, Asp93
**4**	−4.09	Asn51, Asp93, Gly97, Thr184
**5**	−5.07	Asn51, Ser52, Asp93
**6**	−7.88	Asn51, Leu103, Phe138, Trp162
**7**	−9.15	Asn51, Gly97, Asp102, Leu107
**9**	−5.13	Asn51, Asp93, Gly97, Thr184
**10**	−6.76	Asn51, Lys58, Asp93, Gly97, Thr184
**11**	−4.88	Asn51, Ser52, Asp93, Gly97, Thr184
**14**	−7.52	Ser52, Asp93, Gly97, Leu107, Thr184
**15**	−8.92	Asn51, Asp93, Gly97, Thr184
**15′**	−8.09	Asp54, Thr99, Asp102, Asn106, Leu107, Gly132
**16**	−7.42	Met98, Leu103, Trp162
**17**	−6.59	Asn51, Phe138, Tyr139
**19**	−4.66	Asn51, Ser52, Asp93, Thr184
**21**	−4.90	Leu107, Trp162
**22**	−8.87	Asn51, Asp54, Asp57, Leu107, Ile110, Gly135
**23**	−8.66	Asn51, Trp162
**24**	−7.02	Asn51, Asp93
**26**	−6.81	Asn51, Asp93, Gly97, Thr184
**27**	−7.11	Asn51, Leu107, Phe138, Thr184
**29**	−6.88	Asn51, Phe138, Tyr139
**30**	−7.31	Asn51, Phe138, Trp162
**31**	−8.15	Asn51, Ser52, Asp93
**32**	−9.41	Asp54, Lys58, Asp93, Leu107
**33**	−7.93	Asp93, Gly97, Gly135, Thr184
**34**	−8.14	Asn51, Asp93, Leu107
**35**	−7.59	Asn51, Asp93
**37**	−8.11	Asn51, Asp93, Gly97, Leu107, Thr184
**37**	−6.28	Asn51, Lys58
**38**	−8.66	Asn51, Leu107, Gly135, Phe138
**39**	−9.81	Asn51, Asp54, Lys58, Leu107, Gly135
**42**	−8.24	Asn51, Asp93, Gly97, Leu107, Thr184
**44**	−6.96	Asn51, Asp93, Phe138, Thr184
**46**	−7.30	Asn51, Gly135, Thr184
**FJ5 (control)**	−8.27	Ser52, Asp93, Thr184
**Radicicol (control)**	−6.77	Asp93

* Compound numbers from Table 1.

**Table 4:** Docking scores (kcal/mol) and interactions of *E. divinorum* metabolites in the active site of inducible nitric oxide synthase (iNOS).

No. *	Score	Interactions
**1**	−5.07	Arg260, Arg375, Gln381, Arg382
**2**	−3.83	Arg260, Tyr341, Asp376, Arg382
**3**	−4.41	Glu371, Arg375
**4**	−4.51	Arg260, Arg375, Gln381, Arg382
**5**	−4.23	Met114, Arg375
**6**	−5.54	Arg260, Met349, Arg382
**7**	−9.54	Trp188, Asn364, Trp457
**9**	−4.07	Met114, Arg260
**10**	−6.38	Arg260, Met349
**11**	−4.10	Arg260, Arg382
**14**	−6.25	Ile195, Trp366, Glu371, Arg375
**15**	−8.87	Glu371
**15′**	−8.67	Met114, Asn348, Met349, Trp457
**16**	−5.85	Arg260, Met349, Arg382
**17**	−5.39	Met349, Glu371
**19**	−4.27	Arg260
**21**	−4.30	Glu371
**22**	−11.09	Met114, Trp188, Arg193, Asn364, Glu371, Trp457
**23**	−10.19	Trp188, Glu371
**24**	−5.88	Cys194, Ile195
**26**	−6.60	Arg260
**27**	−5.86	Arg193, Glu371
**29**	−5.84	Met349, Asp376
**30**	−6.89	Arg260, Met349
**31**	−7.58	Tyr485
**32**	−8.59	Arg260, Arg375, Gln381, Trp457
**33**	−7.32	Arg193, Arg382
**34**	−7.80	Ile195, Trp366, Glu371,
**35**	−6.15	Glu371, Arg375, Trp457
**37**	−7.87	Trp188, Met349, Asn364, Glu371
**37**	−5.86	Arg260, Met349, Arg382
**38**	−7.74	Met114, Arg375
**39**	−8.71	Met114, Arg193, Ile195, Arg375, Trp457
**42**	−7.98	Trp188, Asn364, Glu371
**44**	−5.99	Ile195, Trp366
**46**	−5.93	Ile195
**MPW (control)**	−6.64	Ile195, Trp366
**1400 W (control)**	−5.78	Asn364, Glu371

* Compound numbers from Table 1.

**Table 5:** Average docking scoring functions (kcal/mol) of the most promising compounds from *E. divinorum* with xanthine oxidase, heat shock protein 90 (Hsp90), and inducible nitric oxide synthase (iNOS).

Molecule Numbers from Table 1	Dock Score			Average Score (kcal/mol)
	1	2	3	
**XO**				
**23**	−8.30	−8.30	−8.30	−8.30
**31**	−7.70	−7.62	−7.62	−7.65
**33**	−7.61	−7.48	−7.49	−7.53
Quercetin	−5.81	−5.82	−5.82	−5.82
Febuxostat	−7.55	−7.53	−7.53	−7.54
**Hsp90**				
**7**	−9.15	−9.01	−9.02	−9.06
**15**	−8.92	−9.00	−8.99	−8.97
**22**	−8.87	−9.76	−9.37	−9.33
**23**	−8.66	−8.63	−8.63	−8.64
**32**	−9.41	−10.17	−10.18	−9.92
**38**	−8.66	−8.11	−8.22	−8.33
**39**	−9.81	−9.74	−9.38	−9.64
FJ5	−8.27	−8.42	−8.71	−8.47
Radicicol	−6.77	−5.87	−5.85	−6.16
**iNOS**				
**7**	−9.54	−9.29	−8.85	−9.23
**15**	−8.87	−8.90	−8.92	−8.90
**15′**	−8.67	−10.09	−9.03	−9.26
**22**	−11.09	−11.42	−11.96	−11.49
**23**	−10.19	−10.21	−9.31	−9.90
**30**	−6.89	−7.12	−6.37	−6.79
**31**	−7.58	−7.34	−6.88	−7.27
**32**	−8.59	−9.74	−8.86	−9.06
**33**	−7.32	−7.83	−7.85	−7.67
**34**	−7.80	−8.10	−8.12	−8.01
**37**	−7.87	−7.97	−8.09	−7.98
**38**	−7.74	−7.59	−7.61	−7.65
**39**	−8.71	−9.29	−9.47	−9.16
**42**	−7.98	−6.93	−7.95	−7.62
MPW	−6.64	−6.76	−6.55	−6.65
1400 W	−5.78	−5.97	−5.29	−5.68

**Table 6:** Drug likeness results of the identified compounds from *E. divinorum*.

Cpd No.	MW	H-Bond		Fraction Csp3	Rotatable Bonds	MR	TPSA	Consensus Log-P	ESOL Log-S	ESOL Class	Violations				
		Acceptors	Donors								Lipinski	Ghose	Veber	Egan	Muegge
**1**	192.17	6	5	0.86	1	40.11	118.22	−1.66	0.53	Highly soluble	0	1	0	0	2
**2**	134.09	5	3	0.5	3	26.05	94.83	−1	0.32	Highly soluble	0	4	0	0	2
**3**	174.15	5	4	0.57	1	38.43	97.99	−1.1	0.23	Highly soluble	0	2	0	0	1
**4**	192.12	7	4	0.5	5	37.47	132.13	−1.51	0.38	Highly soluble	0	2	0	1	1
**5**	170.12	5	4	0	1	39.47	97.99	0.21	−1.64	Very soluble	0	2	0	0	1
**6**	332.26	10	7	0.46	4	71.44	177.14	−1.54	−0.93	Very soluble	1	1	1	1	2
**9**	154.12	4	3	0	1	37.45	77.76	0.74	−2.23	Soluble	0	3	0	0	1
**10**	412.32	13	7	0.46	6	81.64	228.89	−1.98	−0.89	Very soluble	2	1	1	1	4
**11**	154.12	4	3	0	1	37.45	77.76	0.75	−1.89	Very soluble	0	3	0	0	1
**14**	326.3	8	5	0.4	5	77.26	136.68	−0.49	−1.37	Very soluble	0	1	0	1	0
**16**	366.3	11	5	0.42	6	73.65	188.43	−1	−1.59	Very soluble	1	1	1	1	2
**17**	286.23	8	5	0.42	4	63.45	136.68	−0.6	−1.64	Very soluble	0	1	0	1	0
**19**	138.12	3	2	0	1	35.42	57.53	1.05	−2.07	Soluble	0	3	0	0	1
**21**	184.15	5	3	0.12	2	43.79	86.99	0.57	−1.73	Very soluble	0	0	0	0	1
**24**	290.27	6	5	0.2	1	74.33	110.38	0.85	−2.22	Soluble	0	0	0	0	0
**26**	354.31	9	6	0.38	5	83.5	164.75	−0.39	−1.62	Very soluble	1	1	1	1	2
**27**	334.28	8	5	0.19	3	81.22	136.68	0.93	−2.72	Soluble	0	0	0	1	0
**29**	306.27	7	6	0.2	1	76.36	130.61	0.42	−2.08	Soluble	1	0	0	0	1
**30**	386.35	10	5	0.47	7	90.24	155.14	−0.56	−1.23	Very soluble	0	1	1	1	1
**31**	458.37	11	8	0.14	4	112.06	197.37	0.95	−3.56	Soluble	2	0	1	1	3
**33**	442.37	10	7	0.14	4	110.04	177.14	1.3	−3.7	Soluble	1	0	1	1	2
**34**	480.38	13	9	0.29	4	112.18	230.74	−0.93	−2.91	Soluble	2	2	1	1	3
**35**	280.23	7	3	0.15	7	67.01	121.13	0.52	−1.65	Very soluble	0	0	0	0	0
**37**	450.35	12	8	0.25	3	106.21	210.51	−0.43	−2.85	Soluble	2	0	1	1	3
**46**	318.24	8	6	0	1	80.06	151.59	0.79	−3.01	Soluble	1	0	1	1	2

**Table 7:** Pharmacokinetics results of the identified compounds from *E. divinorum*.

Cpd No. 2	Bioavailability Score	GI Absorption	BBB Permeant	Pgp Substrate	CYP Inhibitors					Log-Kp (cm/s)	Alerts		Lead-likeness Violations	Synthetic Accessibility
					1A2	2C19	2C9	2D6	3A4		PAINS	Brenk		
**1**	0.56	Low	No	No	No	No	No	No	No	−9.15	0	0	1	3.34
**2**	0.56	High	No	No	No	No	No	No	No	−8.01	0	0	1	2.27
**3**	0.56	High	No	No	No	No	No	No	No	−8.58	0	0	1	3.77
**4**	0.56	Low	No	No	No	No	No	No	No	−8.69	0	0	1	2.18
**5**	0.56	High	No	No	No	No	No	No	Yes	−6.84	1	1	1	1.22
**6**	0.55	Low	No	No	No	No	No	No	No	−9.33	1	1	0	4.17
**9**	0.56	High	No	No	No	No	No	No	Yes	−6	0	1	1	1.1
**10**	0.11	Low	No	No	No	No	No	No	No	−10.25	0	1	1	4.5
**11**	0.56	High	No	No	No	No	No	No	Yes	−6.39	1	1	1	1.09
**14**	0.56	Low	No	No	No	No	No	No	No	−8.7	0	1	0	4.3
**16**	0.11	Low	No	No	No	No	No	No	No	−8.88	0	1	1	4.17
**17**	0.55	Low	No	No	No	No	No	No	No	−7.98	1	1	0	3.85
**19**	0.85	High	Yes	No	No	No	No	No	No	−6.02	0	0	1	1
**21**	0.55	High	No	No	No	No	No	No	No	−6.81	1	1	1	1.5
**24**	0.55	High	No	Yes	No	No	No	No	No	−7.82	1	1	0	3.5
**26**	0.11	Low	No	No	No	No	No	No	No	−8.76	1	2	1	4.16
**27**	0.56	High	No	Yes	No	No	No	No	No	−7.62	1	1	0	3.77
**29**	0.55	High	No	No	No	No	No	No	No	−8.17	1	1	0	3.53
**30**	0.11	Low	No	No	No	No	No	No	No	−9.45	0	1	1	4.68
**31**	0.17	Low	No	No	No	No	No	No	No	−8.27	1	1	1	4.2
**33**	0.55	Low	No	No	No	No	No	No	No	−7.91	1	1	1	4.16
**34**	0.17	Low	No	No	No	No	No	No	No	−9.22	1	1	1	5.36
**35**	0.56	High	No	No	No	No	No	No	No	−7.65	0	3	0	3.09
**37**	0.17	Low	No	No	No	No	No	No	No	−9	1	1	1	5.09
**46**	0.55	Low	No	No	Yes	No	No	No	Yes	−7.4	1	1	0	3.27

**Table 8:** In vitro antioxidant activities and total phenolic content.

Sample	TPC	DPPH^.^	FRAP
	mg GA/g Extract	IC_50_, µg/mL	mM FeSO_4_/g Extract
*E. divinorum* extract	132.32 ± 4.51	20.44 ± 0.23	24.98 ± 1.28
Quercetin	−	1.07 ± 0.01	24.04 ± 1.23

## Data Availability

All the data are available in the manuscript.
